# Can the Palatability of Healthy, Satiety-Promoting Foods Increase with Repeated Exposure during Weight Loss?

**DOI:** 10.3390/foods6020016

**Published:** 2017-02-22

**Authors:** Katherene O.-B. Anguah, Jennifer C. Lovejoy, Bruce A. Craig, Malinda M. Gehrke, Philip A. Palmer, Petra E. Eichelsdoerfer, Megan A. McCrory

**Affiliations:** 1 Department of Human Ecology, Louisiana Tech University, Ruston, LA 71272, USA; kanguah@latech.edu; 2 Department of Nutrition Science and the Ingestive Behavior Research Center, Purdue University, West Lafayette, IN 47907, USA; 3 School of Public Health, The University of Washington, Seattle, WA 98195, USA; jlovejoy@arivale.com; 4 Institute for Systems Biology, Seattle, WA 98019, USA; 5 Department of Statistics, Purdue University, West Lafayette, IN 47907, USA; bacraig@stat.purdue.edu; 6 The School of Nutrition and Exercise Science, and the Bastyr University Research Institute, Bastyr University, Kenmore, WA 98028, USA; mal.gehrke@gmail.com (M.M.G.); phil@nutritionalternatives.com (P.A.P.); petraelena@gmail.com (P.E.E.); 7 Department of Health Sciences, Programs in Nutrition, Boston University/College of Health & Rehabilitation Sciences: Sargent College, Boston, MA 02215, USA; 8 Boston Nutrition and Obesity Research Center, Boston, MA 02118, USA

**Keywords:** pulses, palatability, repeated exposure, weight loss

## Abstract

Repeated exposure to sugary, fatty, and salty foods often enhances their appeal. However, it is unknown if exposure influences learned palatability of foods typically promoted as part of a healthy diet. We tested whether the palatability of pulse containing foods provided during a weight loss intervention which were particularly high in fiber and low in energy density would increase with repeated exposure. At weeks 0, 3, and 6, participants (*n =* 42; body mass index (BMI) 31.2 ± 4.3 kg/m^2^) were given a test battery of 28 foods, approximately half which had been provided as part of the intervention, while the remaining half were not foods provided as part of the intervention. In addition, about half of each of the foods (provided as part or not provided as part of the intervention) contained pulses. Participants rated the taste, appearance, odor, and texture pleasantness of each food, and an overall flavor pleasantness score was calculated as the mean of these four scores. Linear mixed model analyses showed an exposure type by week interaction effect for taste, texture and overall flavor pleasantness indicating statistically significant increases in ratings of provided foods in taste and texture from weeks 0 to 3 and 0 to 6, and overall flavor from weeks 0 to 6. Repeated exposure to these foods, whether they contained pulses or not, resulted in a ~4% increase in pleasantness ratings. The long-term clinical relevance of this small increase requires further study.

## 1. Introduction

Palatability is defined as “the hedonic evaluation of oro-sensory food cues under standardized conditions” [1]. Taste, odor, appearance, texture, temperature, sound, and trigeminal senses which together constitute flavor are sensory characteristics of a food which people use to assess palatability [2]. The palatability of a food, especially its taste pleasantness, is the most important factor that determines food selection or preference [3,4,5]. It is widely accepted that macronutrient composition influences palatability so that generally foods higher in fat and sugar content have higher palatability [6,7,8]. While foods higher in palatability are consumed in higher amounts in controlled studies, independent of macronutrient composition [6,9,10], community-dwelling individuals tend not to self-select foods that are not well-liked [11].

It is generally believed that foods containing fat, salt, and/or sugar, and which tend to be energy dense, become more appealing with repeated exposure [12,13,14,15], though not all studies show this [16,17,18]. For example, after repeated exposure to a saltier diet, individuals preferred higher levels of salt [14]. In other studies, with repeated exposure to less palatable foods, or to low fat foods, individuals increased their acceptance of those foods [15,17,19,20,21,22,23,24,25] and in some cases the intake of those foods increased sufficiently to match the intake of other foods that were initially well-liked [26,27]. In other cases, though, over time palatability declined for initially liked foods [28,29] or remained unchanged [23]. Possibly, factors such as the particular food, the quantity of the food, the nutrient composition of the food, and/or the initial liking of the food may have contributed to the differences in responses across studies.

Healthy satiety promoting foods which are generally rich in indigestible carbohydrates and moderately high in protein are thought to help regulate body weight and reduce the risk for chronic diseases such as type 2 diabetes, heart disease, and some cancers [30,31,32]. These foods are generally promoted in the US Dietary Guidelines for Americans to help manage and reduce chronic disease risk. However, estimates of non-adherence to such dietary changes for managing chronic diseases are high, between 50% and 80% [33] , with one of the main reasons being that these foods may not be very well-liked [34,35]. Pulses, which are legumes harvested solely for their edible dry grain [36], are examples of healthful foods rich in indigestible carbohydrates and moderately high in protein that have been shown to help with weight control and reduce chronic disease risk. However, pulse consumption in the U.S. is markedly low, with only 8%–30% of US adults consuming pulses over one or two days, and of this proportion, only 35%–42% consume the recommended amount (~1/2 c/day) [37]. Well-controlled, single-meals studies indicate that the palatability or taste pleasantness of pulse containing meals were rated between 8% and 53% lower than non-pulse carbohydrate-based [38,39] or meat-based [40] control meals. Thus, the relatively lower palatability of pulses compared to non-pulse alternatives may be a factor contributing to their low intakes in community-dwelling individuals. 

The purpose of this study was to test the effects of repeated exposure to foods that were provided during a weight-loss intervention, including foods containing pulses, on changes in the palatability ratings of those foods. We hypothesized that due to repeated exposure as part of the intervention study: (1) the palatability of provided foods (containing and not containing pulses) would increase during the intervention and also; (2) for pulse-containing foods, there would be a dose-response effect of pulse on the increase in palatability ratings. The palatability data were collected as part of a weight loss intervention for which the results on weight loss, appetite, dietary intake, and compliance with the intervention will be reported in a future manuscript. Only the results on palatability are reported here.

## 2. Materials and Methods

### 2.1. Study Participants and Design

Participants and study design details will be reported in detail in a future manuscript but are briefly described here. Overweight or obese (body mass index (BMI) 25–40 kg/m^2^) but otherwise healthy, non-smoking men and women aged 21–50 years were recruited for the Beans, Weight Loss, and Lifestyle (Be WeLL) study, a randomized controlled trial conducted at the Bastyr University Research Institute (BURI) in the Seattle, WA area. The goal of the study was to assess dose-response effects of pulse consumption during energy restriction on appetite, body weight and composition, self-selected macronutrient and food intake patterns, cardiovascular and diabetes risk factors, and palatability/taste-preferences for pulses. Regular pulse consumers were excluded from participation; other exclusion criteria included having conditions or taking medications or dietary supplements known to affect energy regulation including taste acuity, and unstable body weight (>5 lbs. change in past three months), and not liking or not being willing to consume any of the foods or food ingredients used in the study. Data analyses were conducted at the Nutrition Science department of Purdue University. The study was approved by the Institutional Review boards at Bastyr University and Purdue University. Written informed consent was obtained from each individual prior to study participation. Forty-nine individuals had enrolled but one dropped from the study prior to randomization. This paper focuses on the results obtained for the palatability outcomes for the 42 participants who completed the six-week intervention. Results on the other outcomes mentioned above will be published in a future manuscript.

**Figure 1:**
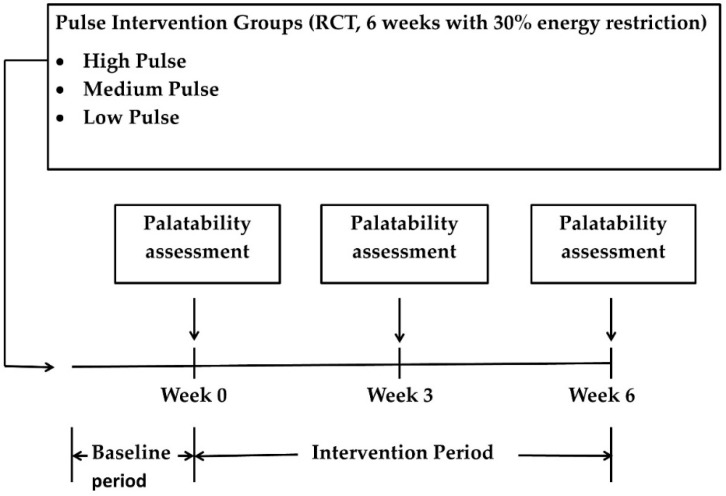
Schematic of Study Design. RCT, randomized controlled trial.

The study design is shown in Figure 1. Palatability assessments using a test battery of foods, some of which had been provided for consumption during the intervention, were performed at weeks 0, 3, and 6. Forty-eight participants were randomly assigned to low (1 tablespoon or 15 mL/day for six days/week), medium (~0.5 cup or 120 mL/day for six days/week to achieve the US Dietary Guidelines recommendation for legume consumption exclusively from pulses [41]), or high pulse consumption groups (1.8 cups or 432 mL/day or 2.7 cups or 600 mL/day for women and men respectively, for six days/week, to achieve the Adequate Intake of dietary fiber exclusively from pulses of 25 and 38 g/day for women and men, respectively [42], for six weeks while undergoing 30% energy restriction. All groups received four food items/day; participants were asked to consume one item each with breakfast, lunch, dinner, and a snack. An energy intake goal and counseling on how to achieve this goal were provided to each participant. Women received 1000 kcal/day (250 kcal per item) and men received 1200 kcal/day (300 kcal per item. These food items accounted for approximately 45%–55% of the target energy intake, depending on individual energy requirements. The pulse containing foods were provided to participants as part of the four food items per day for six days a week. Some or all of the provided study foods contained pulses depending on the intervention group to which the participant belonged. A total of 24 foods were created, each having three recipe versions, containing either no pulses, 1 tablespoon of pulses, or 0.5 cup of pulses. There was a three-week rotating menu and within a given week, each food reappeared on the menu only once. The mean macronutrient distribution of provided food was similar across groups, but individual foods varied from 15% to 35% of energy from protein, 15%–45% of energy from fat, and 30%–65% energy from carbohydrate. A variety of pulses and pulse components were used, including black beans, cannellini beans, chickpeas, chickpea flour, great northern beans, peas, pea hull fiber, and pinto beans. Foods were prepared in the research kitchen at Bastyr University, and packaged for the participants. 

### 2.2. Assessment of Palatability

Participants tasted and rated the test battery of 28 multiple-ingredient, typical American-style foods for pleasantness of appearance, taste, odor, and texture. The test battery (Table 1) included a range of flavors and textures and was balanced with respect to food type (containing pulses or not containing pulses) and exposure type (provided as part of the intervention or not provided as part of the intervention). Foods not provided as part of the intervention and foods not containing pulses served as controls that would allow us to tease out the separate effects of pulses and food provision, and their interaction on changes in palatability ratings during the intervention. Most of the foods were prepared in the research kitchen at Bastyr University by following a recipe, but some were purchased off the shelf and required no preparation other than heating for some (e.g., peas, Amy’s Enchilada Bowl, applesauce, asparagus). 

The order of food tasting was randomized, with each participant assigned to one of three possible orders, and each participant tasted the foods in the same order for each of the three test sessions (see Supplementary Materials Table S1). Inadvertently, seven study participants tasted 2 to 4 foods out of order and this alteration was examined in the statistical analysis but had no effect on the results, as described below. During tasting sessions, one bite-sized piece of each food was presented in a condiment cup and served at an appropriate temperature for consumption (i.e., cold, room temperature, warm, or hot, depending on the food). Participants were given 1 minute to taste and rate each food, and take a sip of water prior to the presentation of the next food. Palatability was assessed using a standard nine-point scale. The scale was anchored at 1 and 9, respectively, with “extremely unpleasant” and “extremely pleasant”. 

### 2.3. Data Analysis

All statistical analyses were performed by using SAS software (version 9.3, SAS Institute Inc., Cary, NC, USA) and Statistical Package for Social Sciences (IBM SPSS Statistics, version 20, SPSS Inc., Armonk, NY, USA). Participant characteristics are expressed as mean ± SD (standard deviation); all other variables are expressed as estimate ± SE (standard error). Differences in participant characteristics at baseline among treatment groups were analyzed by using one-way analysis of variance (ANOVA) with post-hoc analysis of paired comparisons using Tukey’s HSD (honest significant difference) test. For each of the 28 food items, a flavor score was calculated as the average of the appearance, taste, odor, and texture pleasantness ratings. Thus, five palatability variables (taste, appearance, odor, texture, and flavor pleasantness) were included as outcomes. Analyses focused on each participant’s aggregated palatability ratings, which were computed by averaging the ratings for foods in the same category (provided as part of the intervention/pulse, provided as part of the intervention/not-pulse, not provided as part of the intervention/pulse or not provided as part of the intervention/not-pulse) for weeks 0, 3, and 6. Cronbach’s analysis showed that this approach was reliable (Cronbach’s α values showed a high degree of correlation between ratings from the same category, ranging from 0.78 to 0.90). 

Repeated measures ANOVA on each aggregated palatability score was performed, with exposure type (provided or not provided as part of the intervention), food type (containing pulses or not containing pulses), pulse amount (low, medium, high), and time (week 0, 3, and 6) as the fixed factors including all possible two, three, and four-way interaction effects, by using a mixed model (proc mixed in SAS). A linear mixed model was used to account for possible correlation among the 12 observations per participant and various correlation structures were considered. The model with the lowest BIC (Bayesian Information Criteria, a model selection criteria that balances complexity and fit) was selected. To determine if the seven participants that rated between two and four foods out of order from their assigned randomized tasting order had affected the results, the analysis was performed both with and without the data for these visits included, and there was no qualitative difference in the results. Therefore, we present the results in which data for all visits for each participant are included. A *p*-value of <0.05 was considered significant.

## 3. Results

### 3.1. Characteristics of the Participants 

There were 42 participants (13, 16, and 13 in the low, medium, and high pulse groups, respectively). The three pulse consumption groups did not differ at baseline in sex distribution (92%, 81%, and 85% of participants in the low, medium, and high pulse groups, respectively, were females), BMI (low pulse group 31.0 ± 4.2, medium pulse group 32.3 ± 5.1, and high pulse group 30.0 ± 3.1 kg/m^2^), or age (40 ± 7, 38 ± 7, and 40 ± 7 years for low, medium, and high pulse groups, respectively). 

### 3.2. Main Effects of Exposure Type, Food Type, Pulse Treatment Level, and Time 

Values for palatability ratings ranged from a low of 6.4 ± 0.3 to a high of 7.5 ± 0.2 and varied similarly across taste, odor, texture, appearance, and flavor variables (Table 2). 

There were no differences in palatability ratings by food type (pulse, not pulse), pulse treatment group (low, medium, high), or time (week, 0, 3, 6) (*p* > 0.05). However, there was a significant main effect of exposure type, such that provided foods were rated about 3.4% lower than foods that were not provided as part of the intervention across all time points for most variables (taste, appearance, texture, flavor; *p* = 0.0048–<0.0001) with the exception of odor (*p* = 0.241).

### 3.3. Interaction Effects

**Figure 2:**
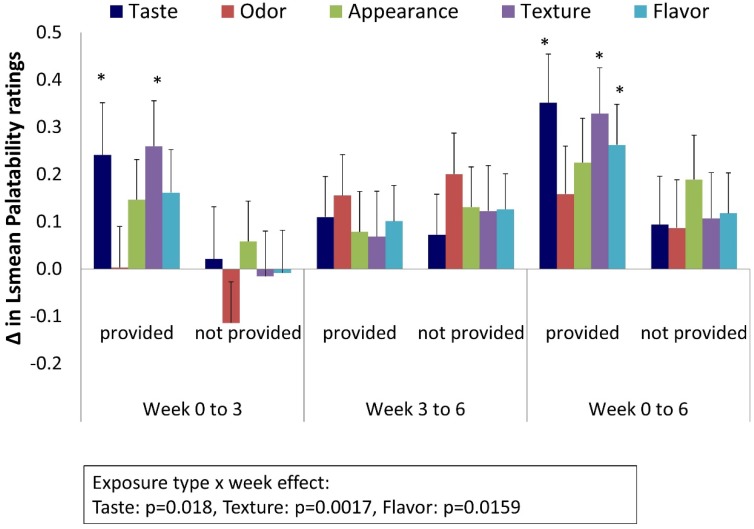
Changes in palatability ratings over the six-week pulse intervention in provided and not provided foods. Changes were calculated by subtracting the adjusted mean at the earlier time point from the later time point. Values are presented as least square (LS) mean ± SEM (standard error of the mean). * There were significant week by exposure type interaction effects for taste (*p =* 0.018), texture (*p =* 0.002), and flavor (*p =* 0.016) (see Table 2 for *p*-values for changes from weeks 0 to 3 and weeks 0 to 6).

Changes in the palatability ratings over time by exposure type are shown in Figure 2 and Table 2. For most variables, palatability ratings increased over time, with the exception of odor, texture, and flavor from weeks 0 to 3 for foods not provided during the intervention. There was, a significant week by exposure type effect for taste (*p* = 0.018), texture (*p* = 0.002), and flavor (*p* = 0.016). Specifically, in provided foods, but not in foods that were not provided, changes in taste and texture ratings were significant from weeks 0 to 3 (*p* ≤ 0.031), and changes in the taste, texture, and flavor were significant from weeks 0 to 6 (*p* = 0.001–0.003). There were no significant changes over time in palatability ratings for any of the variables from weeks 3 to 6. Thus, overall, there was an increase in the ratings for taste, texture, and flavor from week 0 to week 6 in provided foods with an average increase of 0.3 ± 0.1 points on the nine-point hedonic scale, whereas the average change over this same time period in these variables in the not provided foods was 0.1 ± 0.1 points. There were no significant interactions with pulse type or treatment.

## 4. Discussion

Previous work has shown that in the US, pulse consumption is well below recommended intake amounts and that pulses are not well-liked compared to carbohydrate-based control foods. Since repeated exposure to foods rich in fat, sugar, and salt often increases preference for those foods, we investigated whether repeated exposure to healthier foods, including those containing pulses, which are high in fiber, and low in fat and sugar, would likewise result in increased palatability ratings over time during a weight loss intervention. We found that some components of palatability (taste, texture, and overall flavor pleasantness), but not others (appearance and odor), for foods which had been provided during the intervention increased, but this did not occur for foods that were not provided during the intervention. Thus, merely exposing individuals to these foods, regardless of whether they contained pulses or not, resulted in increased ratings of taste, texture, and flavor over six weeks, the latter of which encompasses multiple sensory qualities. Taken together with previous work, our results suggest that it is possible for foods which are often promoted as components of healthful diets to prevent and treat obesity and chronic disease, including pulses, to become more preferred over time with repeated exposure. Additional studies are needed to determine if the magnitude of increase observed in this study (4%) will translate to a greater selection of these foods in everyday life, whether combined or not with other educational and lifestyle factors. 

We found that from week 0 to 3, for provided foods, taste and texture pleasantness increased and continued to increase through the end of the six-week intervention, by which time flavor pleasantness had also increased. In contrast, the palatability ratings of foods that had not been provided did not change over time. Most of the increases in the palatability of provided foods occurred in the first three weeks of the intervention, which is within the time frame that increases have been observed in other studies (three days to ~10 weeks) [19,20,21,22,23,24,25]. Several studies have shown that with repeated exposure, individuals learned to accept previously disliked foods [19,20,21,22,23,24], although not all studies have shown this [16,17,18]. Some researchers have reported increases in liking or acceptance of fruits and vegetables which were previously disliked of 15% to 53% from baseline. Accordingly, in these studies, intake of the fruits and vegetables increased by 21% to 350%. Yet in some studies, although liking for initially less liked or low palatability foods did not improve with repeated exposure, intakes of the foods increased nonetheless [17,26]. In another study, the opposite occurred: participants increased their acceptance of goat milk (by about 8%) with repeated exposure for six days, but this increase was not sufficient to cause an overall product acceptance [23]. These discordant findings could possibly be attributed to the wide variation across studies in repeated exposure duration (three days to 10 weeks), or to different test foods or control foods used. In the present study, palatability for provided foods increased by approximately 0.3 points (4%), yet it is uncertain if this increase is enough to sustain self-selected intake under natural conditions. Alternatively, the lack of decrease in palatability of provided foods over six weeks of repeated consumption, regardless of whether they contained pulses, may be considered as signifying a strong potential for long-term consumption, as in another, similar study on Australian sweet lupin kernel fiber [43].

Healthy high fiber, low fat foods are promoted for weight loss and prevention of chronic diseases, but people do not generally adhere long-term to such dietary regimens. Some of the core explanations for the low adherence to healthy diets include disliking of the taste, lack of meal preparation or recipe skills, poor nutrition knowledge of health benefits, difficulty making changes to poor dietary habits, and others [34,35]. In one review [34], the authors proposed a number of potentially beneficial interventions that could be used in practice to improve adherence (e.g., nutritional tools, feedback, telephone follow up, etc.), though they also stated that the studies they considered did not necessarily combine these different approaches in the same fashion, which made comparisons across studies difficult. In another review [6], Drewnowski concluded that taste, among other factors, should be taken into account in dietary interventions aimed at improving diet quality. A factor to consider, however, is that if repeated exposure does result in higher intakes, will intake be so high that weight loss efforts are countered? In one study [44], repeated exposure to high energy snack foods reduced sensory specific satiety and increased intake, showing the potential to thwart weight loss efforts. Similar findings were reported in another study in obese, but not non-obese, participants [45]. Alternatively, some studies indicate that with repeated exposure, participants are able to learn about the satiating capacity of a food through its energy content rather than by the sensory characteristics, and hence increase intake of low energy foods while decreasing intake of high energy foods [46,47]. Similar findings were reported in non-obese, but not obese, participants fed a 300 kcal snack per day for two weeks compared to the same snacks of lower or no energy [48]. Thus, it can be speculated that consumption of healthier foods may improve with repeated exposure, but that intake might not be so much as to offset weight loss efforts in a lifestyle intervention because participants may learn to associate intakes with energy content rather than sensory qualities. 

Pulse foods in our study were rated as moderately pleasant (~7 points on the nine-point scale) for palatability at baseline, which is slightly higher than ratings of pulse foods in other studies of ~6 (also on a nine-point scale) [38], 59 on a 100 mm visual analogue scale (VAS) (after correction for reverse scoring) [40], and much higher than those in another study of 0.7 on a five-point scale [39]. Thus, in our study palatability ratings for pulses were higher than previously reported, possibly because we used a variety of pulses rather than just one pulse type, and because participants tasted pulses incorporated into recipes rather than pulses alone. Since the ratings for pulse-containing foods in our study were somewhat high to begin with, this could have accounted for why there was very little increase in palatability rating over the course of the intervention. Though the change in palatability we observed in this six-week study may not be enough to cause a change in behavior by itself, future studies should examine the combined effects of compulsory repeated exposure and behavior change tools, such as education on the health benefits of consuming pulses, different and easy ways for preparing pulses, and how to gradually and easily transition to including pulses more frequently in everyday meals through the use of support of family and friends on subsequent self-selection of pulses and other healthful foods [49]. 

Contrary to our hypothesis, we did not observe a dose-response effect of pulse amount during the intervention on palatability ratings with repeated exposure. One explanation for this result may be that participants in the high pulse group became habituated [50] with too much pulses and that this introduced a monotony effect which could decrease acceptance [51,52]. Alternatively, mere exposure may have the overriding influence over the specific dose. In a study in which children were repeatedly exposed to bell peppers ad libitum, repeatedly inviting the children to taste the peppers regardless of how much they ate was a good strategy for promoting liking and intake of the peppers [19]. Similarly in a cross-sectional study of older low fruit consumers, repeated exposure for five weeks was enough to increase fruit intake, and there was no added benefit of extra fruit provision [18]. In another study, however, self-reported food liking was significantly reduced after two weeks of daily exposure to a 300 kcal snack compared to 0 and 100 kcal portions of the same snack [48]. Based on the first two studies [18,19] and our present results, it appears that exposure, but not the amount of exposure, is enough to increase palatability, whereas a higher amount of exposure could possibly decrease liking. 

One of the major strengths of this study was our design which allowed us to determine the separate effects of repeated exposure to both pulse and non-pulse foods on palatability using non-exposure and foods not containing pulses as controls. Also, the order for rating the palatability for study foods was randomized and remained mostly consistent within the same participant across time points. This helped to minimize differences in ratings within the same subjects which could be brought about by the order in which foods were tasted. Furthermore, we had a three-week rotating menu for the foods we provided to study participants during the weight loss intervention and reused any given food only once within a given week to help maintain interest. Participants were allowed to self-select foods in much of their regimen, which created variety in the diet overall. Our study was not without limitations, however. During the taste rating procedure, we did not use nose clips so our taste assessments may have been confounded by both ortho- and retronasal olfaction [53]. We also used the average palatability scores of several foods combined, which made our analyses even more complex. In addition, the method we used to assess palatability (taste test) may not be a reliable predictor of intake differences among individuals, as some have argued that taste pleasantness tests are better predictors of consumption differences within the same individual than among different individuals [54], whereas taste-and-spit pleasantness ratings better predict consumption differences between individuals [55]. Furthermore, while individuals seek to make dietary changes during a weight loss intervention, it is important to note that the effects of repeated exposure on palatability may differ during weight loss compared with weight stability. Thus, weight loss may be seen as a confounding factor in our study, and the inclusion of a control group which was not undergoing weight loss would have allowed us to discern this potential difference.

## 5. Conclusions

Repeated exposure to healthier foods relatively high in fiber, moderate in protein, and low in fat and sugar, which had been provided during a six-week weight loss intervention, resulted in a ~4% increase in pleasantness ratings whether they contained pulses or not. The long-term clinical relevance of this small increase in palatability requires further study.

## Figures and Tables

**Table 1:** The 28-food item taste test battery for study participants classified according to exposure and food types.

	Food Type	
**Exposure Type**	Containing pulses	Not containing pulses
	Snack mix plain (chickpeas)	Snack mix sweet and salty
	Thai dip with pita (pinto beans)	Corn muffin
	Harvest soup (lentils)	BBQ bake
Provided as	Quesadilla (pinto beans)	Apricot bulgur
part of intervention	Egg casserole (great northern beans)	Vegetable soup
	Peanut butter energy bar (chickpea flour, pea hull fiber)	Brownie
	Chocolate mint pudding (pea hull fiber)	Vanilla pudding
	Peas Crackers and hummus (chickpeas)	Applesauce Asparagus
Not	Santa Fe stew (cannellini beans)	Peanut butter pretzels
provided as part	Bean salad (black beans)	Luna bar
of intervention	Curry (chickpeas)	Pasta marinara
	Amy’s Enchilada Bowl (black beans)	Scalloped corn
		Cashew pudding Carrot muffin

BBQ, barbeque.

**Table 2:** Taste, odor, appearance, texture, and flavor pleasantness ratings by exposure type (provided or not provided as part of the intervention), food type, and pulse treatment groups and week of intervention (least square mean ± standard error of the mean).

	Pulse Treatments								
	Low			Medium			High		
	Week 0	Week 3	Week 6	Week 0	Week 3	Week 6	Week 0	Week 3	Week 6
**Taste ^+^**									
Provided									
Pulse food	6.8 ± 0.2	7.1 ± 0.2	7.2 ± 0.2	6.7 ± 0.2	7.1 ± 0.2	7.2 ± 0.2	7.2 ± 0.2	7.0 ± 0.2	7.1 ± 0.2
Not pulse food	6.5 ± 0.2	6.7 ± 0.2	6.9 ± 0.2	6.9 ± 0.2	7.3 ± 0.2	7.4 ± 0.2	6.6 ± 0.2	6.8 ± 0.2	7.0 ± 0.2
Not provided *									
Pulse food	7.2 ± 0.2	7.0 ± 0.2	7.1 ± 0.2	7.1 ± 0.2	7.3 ± 0.2	7.1 ± 0.2	7.2 ± 0.2	7.0 ± 0.2	7.2 ± 0.2
Not pulse food	7.0 ± 0.2	7.1 ± 0.2	7.4 ± 0.2	7.1 ± 0.2	7.5 ± 0.2	7.4 ± 0.2	7.3 ± 0.2	7.2 ± 0.2	7.2 ± 0.2
**Odor ^†,ɸ^**									
Provided									
Pulse food	7.0 ± 0.2	7.1 ± 0.2	7.1 ± 0.2	6.8 ± 0.2	7.1 ± 0.2	7.2 ± 0.2	6.9 ± 0.2	6.8 ± 0.2	7.1 ± 0.2
Not pulse food	6.9 ± 0.2	6.8 ± 0.2	6.9 ± 0.2	6.9 ± 0.2	7.1 ± 0.2	7.3 ± 0.2	7.0 ± 0.2	6.6 ± 0.2	6.9 ± 0.2
Not provided									
Pulse food	7.1 ± 0.2	6.8 ± 0.2	6.9 ± 0.2	6.8 ± 0.2	7.0 ± 0.2	7.0 ± 0.2	7.0 ± 0.2	6.6 ± 0.2	7.0 ± 0.2
Not pulse food	7.1 ± 0.2	7.1 ± 0.2	7.3 ± 0.2	7.1 ± 0.2	7.4 ± 0.2	7.5 ± 0.2	7.2 ± 0.2	6.8 ± 0.2	7.2 ± 0.2
**Appearance ^∆^**									
Provided									
Pulse food	7.0 ± 0.2	7.2 ± 0.2	7.0 ± 0.2	6.5 ± 0.2	6.7 ± 0.2	6.8 ± 0.2	6.9 ± 0.2	6.8 ± 0.2	7.1 ± 0.2
Not pulse food	6.6 ± 0.2	7.0 ± 0.2	7.0 ± 0.2	6.5 ± 0.2	7.0 ± 0.2	6.9 ± 0.2	6.8 ± 0.2	6.7 ± 0.2	6.9 ± 0.2
Not provided ^ø^									
Pulse food	7.1 ± 0.2	6.9 ± 0.2	7.2 ± 0.24	6.6 ± 0.2	7.0 ± 0.2	7.0 ± 0.2	7.1 ± 0.2	6.9 ± 0.2	7.2 ± 0.2
Not pulse food	7.0 ± 0.2	7.2 ± 0.2	7.1 ± 0.24	6.8 ± 0.2	7.1 ± 0.2	7.2 ± 0.2	7.1 ± 0.2	7.0 ± 0.2	7.1 ± 0.2
**Texture ^₭,$^**									
Provided									
Pulse food	6.4 ± 0.3^a^	7.0 ± 0.3	6.9 ± 0.3	6.6 ± 0.2	6.9 ± 0.2	7.0 ± 0.2	6.9 ± 0.3	6.7 ± 0.3	6.9 ± 0.3
Not pulse food	6.4 ± 0.3^ab^	6.5 ± 0.3	6.7 ± 0.3	6.6 ± 0.2	7.0 ± 0.2	7.1 ± 0.2	6.3 ± 0.3^a^	6.7 ± 0.3	6.6 ± 0.3
Not provided ^₮^									
Pulse food	7.3 ± 0.3^c^	7.1 ± 0.3	7.1 ± 0.3	7.0 ± 0.2	7.0 ± 0.2	7.2 ± 0.2	7.2 ± 0.3^bc^	7.0 ± 0.3	7.2 ± 0.3
Not pulse food	6.9 ± 0.3	7.1 ± 0.3	7.3 ± 0.3	6.9 ± 0.2	7.2 ± 0.2	7.3 ± 0.2	7.2 ± 0.3^b^	7.1 ± 0.3	7.1 ± 0.3
**Flavor ^α,λ^**									
Provided									
Pulse food	6.8 ± 0.2	7.1 ± 0.2	7.1 ± 0.2	6.7 ± 0.1	7.0 ± 0.2	7.0 ± 0.2	7.1 ± 0.2	6.9 ± 0.2	7.2 ± 0.2
Not pulse food	6.6 ± 0.2	6.8 ± 0.2	6.9 ± 0.2	6.7 ± 0.1	7.1 ± 0.2	7.2 ± 0.2	6.7 ± 0.2	6.7 ± 0.2	6.9 ± 0.2
Not provided ^γ^									
Pulse food	7.2 ± 0.2	6.9 ± 0.2	7.1 ± 0.2	6.9 ± 0.1	7.1 ± 0.2	7.1 ± 0.2	7.0 ± 0.2	6.8 ± 0.2	7.1 ± 0.2
Not pulse food	7.0 ± 0.2	7.1 ± 0.2	7.3 ± 0.2	7.0 ± 0.1	7.3 ± 0.2	7.4 ± 0.2	7.2 ± 0.2	7.0 ± 0.2	7.2 ± 0.2

In the same column and variable, values with different letters are significantly different. * Different from provided (main effect) *p =* 0.0048. ^+^ Week × provided/not provided interaction effect, *p =* 0.018; ^a^ week 0: not provided > provided *p <* 0.001, ^b^ provided: week 0 < week 3 *p =* 0.0314, ^c^ provided: week 0 < week 6 *p =* 0.0009. ^†^ Treatment × week interaction effect, *p =* 0.0395; ^a^ high: week 0 > week 3 *p =* 0.0242, ^b^ high: week 3 < week 6 *p =* 0.0146, ^c^ medium: week 0 < week 3 and week 0 < week 6 *p =* 0.0400. ^ɸ^ Provided/not provided × pulse/not pulse interaction effect, *p =* 0.0058; ^a^ not provided/not pulse> provided/not pulse *p =* 0.0056, b not provided/not pulse > not provided/pulse *p =* 0.0035. ^ø^ Different from provided (main effect) *p =* 0.0003. ^∆^ Marginal treatment × pulse/not pulse effect *p =* 0.0510. ^₮^ Different from provided (main effect) *p <* 0.0001. ^₭^ Week × provided/ not provided interaction effect *p =* 0.0017; ^a^ week 0, 3, and 6: provided < not provided *p <* 0.0001, *p =* 0.0006 and *p <* 0.0001, respectively, ^b^ provided: week 0 < week 3 and week 0 < week 6 *p =* 0.0080 and 0.0013, respectively. ^$^ Treatment × week × provided/not provided × pulse/not pulse interaction effect *p =* 0.0221. ^γ^ Different from provided (main effect) *p =* 0.0002. ^α^ Week × provided/ not provided interaction effect *p =* 0.0159; ^a^ week 0, 3, and 6: provided < not provided *p <* 0.0001, *p =* 0.0340, *p =* 0.0137, respectively, ^b^ provided: week 0 < week 6 *p =* 0.0028. ^λ^ Marginal week effect *p =* 0.0516.

## Supplementary Materials

The following are available online at www.mdpi.com/2304-8158/6/2/16/s1, Table S1: Randomized orders for tasting of the 28-food item test battery.
