# Supplementary material for: Can the Palatability of Healthy, Satiety-Promoting Foods Increase with Repeated Exposure during Weight Loss?
Source: Foods. 2017 Feb 22;6(2):16. doi: 10.3390/foods6020016 (PMC5332909; doi:10.3390/foods6020016)
Supplement: Supplementary file 1 [file foods-06-00016-s001.pdf]

Supplementary Materials: Can the Palatability of Healthy, Satiety-Promoting Foods Increase with Repeated Exposure during Weight Loss?

Katherene O.-B. Anguah, Jennifer C. Lovejoy, Bruce A. Craig, Malinda M. Gehrke, Philip A. Palmer, Petra E. Eichelsdoerfer and Megan A. McCrory

Table S1. Randomized orders for tasting of the 28-food item test battery.

**Order 1**		**Order 2**		**Order 3**	
Food #	Food Name	Food #	Food Name	Food #	Food Name
13	Brownie	11	Apricot Bulgur	5	Brunch Casserole
11	Apricot Bulgur	13	Brownie	3	Harvest Soup
23	Peanut Butter Pretzels	23	Peanut Butter Pretzels	13	Brownie
12	Veggie Soup with Turkey Bacon	3	Harvest Soup	21	Applesauce
16	Hummus and Crackers	12	Veggie Soup with Turkey Bacon	4	Quesadilla
15	Peas	2	Thai Dip with Pita	18	Black Bean Salad
19	Chickpea Curry	14	Vanilla Pudding	9	Corn Muffin
24	Luna Bar	18	Black Bean Salad	11	Apricot Bulgur
21	Applesauce	19	Chickpea Curry	8	Sweet and Salty Snack Mix
26	Scalloped Corn	28	Carrot Muffin	16	Hummus and Crackers
9	Corn Muffin	10	BBQ Bake	12	Veggie Soup with Turkey Bacon
2	Thai Dip with Pita	5	Brunch Casserole	6	Peanut Butter Choc. Chip Energy Bar
6	Peanut Butter Choc. Chip Energy Bar	22	Asparagus	28	Carrot Muffin
4	Quesadilla	6	Peanut Butter Choc. Chip Energy Bar	26	Scalloped Corn
18	Black Bean Salad	1	Snack Mix (Plain)	2	Thai Dip with Pita
14	Vanilla Pudding	21	Applesauce	15	Peas
28	Carrot Muffin	25	Pasta Marinara	22	Asparagus
3	Harvest Soup	20	Enchilada Bowl	14	Vanilla Pudding
22	Asparagus	16	Hummus and Crackers	27	Cashew Pudding
17	Santa Fe Stew	4	Quesadilla	25	Pasta Marinara
5	Brunch Casserole	8	Sweet and Salty Snack Mix	10	BBQ Bake
20	Enchilada Bowl	9	Corn Muffin	20	Enchilada Bowl
25	Pasta Marinara	17	Santa Fe Stew	23	Peanut Butter Pretzels
7	Chocolate Mint Pudding	24	Luna Bar	1	Snack Mix (Plain)
8	Sweet and Salty Snack Mix	15	Peas	19	Chickpea Curry
1	Snack Mix (Plain)	26	Scalloped Corn	7	Chocolate Mint Pudding

10	BBQ Bake	7	Chocolate Mint Pudding	17	Santa Fe Stew
27	Cashew Pudding	27	Cashew Pudding	24	Luna Bar

#, arbitrary number assigned to each food item for easy identification; BBQ, barbeque.
